# The DM-scope registry: a rare disease innovative framework bridging the gap between research and medical care

**DOI:** 10.1186/s13023-019-1088-3

**Published:** 2019-06-03

**Authors:** Marie De Antonio, Céline Dogan, Ferroudja Daidj, Bruno Eymard, Jack Puymirat, Jean Mathieu, Cynthia Gagnon, Sandrine Katsahian, the Filnemus Myotonic Dystrophy Study Group, Marie Christine Arne Bes, Shahram Attarian, Anne-Catherine Aube-Nathier, Frédérique Audic, Nathalie Bach, Christine Barnerias, Anne-Laure Bedat-Millet, Anthony Behin, Remi Bellance, Rabah Benyaou, Véronique Bombard, Françoise Bouhour, Celia Boutte, François Boyer, Claude Cances, Brigitte Chabrol, Jean-Baptiste Chanson, Françoise Chapon, Raphaële Chasseriau, Pascal Cintas, Ana-Maria Cobo, Vanessa Colombert, Marie-Carmen Cruz, Jean-Marie Cuisset, Romain Deschamps, Isabelle Desguerre, Julien Durigneux, Fanny Duval, Caroline Espil, Catherine Fafin, Léonard Feasson, Mélanie Fradin, Alain Furby, Alice Goldenberg, Sarah Grotto, Karima Ghorab, Lucie Guyant-Marechal, Delphine Heron, Arnaud Isapof, Agnes Jacquin-Piques, Hubert Journel, Pascal Laforet, Emmanuelle Lagrue, Cécile Laroche-Raynaud, Vincent Laugel, Françoise Lebeau, Armelle Magot, Véronique Manel, Michèle Mayer, Sandra Mercier, Dominique Menard, Maud Michaud, Marie-Christine Minot, Raul-Juntas Morales, Aleksandra Nadaj-Pakleza, Jean-Baptiste Noury, Laurent Pasquier, Sybille Pellieux, Yann Pereon, Julie Perrier, Sylviane Peudenier, Marguerite Preudhomme, Jean Pouget, Susana Quijano-Roy, Sylvie Ragot-Mandry, Christian Richelme, François Rivier, Pascal Sabouraud, Sabrina Sacconi, Emmanuelle Salort-Campana, Catherine Sarret, Stéphane Schaeffer, Guilhem Sole, Tanya Stojkovic, Frédéric Taithe, Hervé Testard, Vincent Tiffereau, Andoni Urtizberea, Catherine Vanhulle, Christophe Vial, Ulrike Walther-Louvier, Fabien Zagnoli, Dalil Hamroun, Guillaume Bassez

**Affiliations:** 1 Neuromuscular Reference Center, AP-HP, Pitié-Salpêtrière Hospital, Paris, France; 2 INSERM U1138, Centre de Recherche des Cordeliers, Sorbonne University, Paris Descartes University, Paris, France; 3 Human Genetics, CHUQ/CHUL, Québec, Canada; 4 Groupe de recherche interdisciplinaire sur les maladies neuromusculaires (GRIMN), CIUSSS du Saguenay-Lac-St-Jean, Québec, Canada; 5 Centre de recherche Charles-Le-Moyne-Saguenay-Lac-St-Jean sur les innovations en santé (CR-CSIS), Faculté de médecine et des sciences de la santé, Université de Sherbrooke, Québec, Canada; 6 Unit of Epidemiology and Clinical Research, AP-HP, Georges-Pompidou Hospital, Paris, France; 7 University Institute of Clinical Research, CHU, Montpellier, France; 8 INSERM, Research Center in Myology, Sorbonne University, Paris, France

**Keywords:** Myotonic dystrophy, Rare disease registry, Platform, Medical care, Research

## Abstract

**Background:**

The relevance of registries as a key component for developing clinical research for rare diseases (RD) and improving patient care has been acknowledged by most stakeholders. As recent studies pointed to several limitations of RD registries our challenge was (1) to improve standardization and data comparability; (2) to facilitate interoperability between existing RD registries; (3) to limit the amount of incomplete data; (4) to improve data quality. This report describes the innovative concept of the DM-Scope Registry that was developed to achieve these objectives for Myotonic Dystrophy (DM), a prototypical example of highly heterogeneous RD. By the setting up of an integrated platform attractive for practitioners use, we aimed to promote DM epidemiology, clinical research and patients care management simultaneously.

**Results:**

The DM-Scope Registry is a result of the collaboration within the French excellence network established by the National plan for RDs. Inclusion criteria is all genetically confirmed DM individuals, independently of disease age of onset. The dataset includes social-demographic data, clinical features, genotype, and biomaterial data, and is adjustable for clinical trial data collection. To date, the registry has a nationwide coverage, composed of 55 neuromuscular centres, encompassing the whole disease clinical and genetic spectrum. This widely used platform gathers almost 3000 DM patients (DM1 *n* = 2828, DM2 *n* = 142), both children (*n* = 322) and adults (*n* = 2648), which accounts for > 20% of overall registered DM patients internationally. The registry supported 10 research studies of various type i.e. observational, basic science studies and patient recruitment for clinical trials.

**Conclusion:**

The DM-Scope registry represents the largest collection of standardized data for the DM population. Our concept improved collaboration among health care professionals by providing annual follow-up of quality longitudinal data collection. The combination of clinical features and biomolecular materials provides a comprehensive view of the disease in a given population. DM-Scope registry proves to be a powerful device for promoting both research and medical care that is suitable to other countries. In the context of emerging therapies, such integrated platform contributes to the standardisation of international DM research and for the design of multicentre clinical trials. Finally, this valuable model is applicable to other RDs.

## Background

Over the last few years, several international initiatives have aimed to organize clinical research, patient care and health planning in the field of rare diseases (RD) [1–5]. The lack of relevant knowledge and experience concerning many RDs requires better cooperation and infrastructure. A critical step focuses, as a priority, on Rare Disease Registries (RDRs). Indeed, most stakeholders consider registries to be a strategic tool to develop research and improve knowledge in the field of RDs. The European Platform for RDRs project (EPIRARE) has addressed issues associated with the registration of RDs. This project overviewed the current situation and experience of national RDRs in Europe [6, 7]. The study was used to classify RDRs [8], assess and characterize their quality [9], resulting in a set of core recommendations for RD patient registration and data collection [10–12].

The DM-Scope registry was created in 2008, at the beginning of the European initiatives [1] in response to the complex clinical and genetic characteristics of Myotonic Dystrophy (DM), a prototypical example of highly heterogeneous RD. At this time, the knowledge of underlying molecular mechanisms was growing. However, the high variability of clinical features in DM created particular challenges in the design of clinical studies and for health management.

DM encompasses two rare genetic diseases belonging to the family of neuromuscular disorders: Myotonic Dystrophy type 1 (DM1) and Myotonic Dystrophy type 2 (DM2). DM1 is the most common adult muscular dystrophy with a prevalence of about 6.5/100000 [13]. DM2 is more or less prevalent than DM1 according to certain countries [14, 15]. Both types are recognisable by their multisystemic involvement [14, 16] and their high clinical inter- and intra-individual variability due to the nature of the underlying atypical mutation i.e. an unstable nucleotide repeat expansion [16–22]. The clinical spectrum is particularly large in DM1 with an anticipation phenomenon between generations and is expressed in five different clinical forms, ranging from neonatal to late adult-onset forms [23]. The correlation between phenotype and molecular mechanisms is better understood [24–26] but the part of genetic or epigenetic factors causing the variability of DM1 multisystemic involvement in a given individual remains to be better defined [27–36].

In the last few decades, the increasing understanding of DM pathogenesis has led to the development of several therapeutic approaches [37–40]. It is therefore necessary to gather relevant clinical, genetic and epidemiological data in a large DM population. The evaluation of the efficiency of new therapies requires identification of endpoint measures and informative biomarkers to assess their effect on disease progression. Of note, despite previous studies, the natural history of DM has not yet been fully described [41–43].

During the last decade, we developed, in accordance with European recommendations, a shareable and interoperable framework (DM-Scope system) to promote a quality multicentre collection of data from a large cohort of French DM patients. As DM patients cognitive impairment limits their own contribution to data collection, we developed an integrative platform to promote the contribution of practitioners from the French RD Reference centres [44]. Based on a reciprocal collaboration, DM-Scope system standardizes optimal data collection and facilitates the use of collected data for both medical care and clinical research.

The primary purposes of this paper are to present: (1) the concept of the DM-Scope registry to overcome at most the challenges of RDRs; (2) the innovative tools of the integrative platform; (3) the clinical network activity; (4) the main characteristics of the DM cohorts i.e. demographic-social conditions, professional categories, clinical forms, and mortality.

## Material and methods

### DM-scope registry concept

The DM-Scope registry was developed in France in 2008. The main objective was to increase the epidemiological knowledge in DM, to harmonize patients medical follow-up, and to facilitate selection and enrolment of DM patients in clinical trials, particularly in a multicentre setting. The multi-sites implementation benefited from the national plan for RDs initiated in 2005 in France with the designation of 131 centres of reference at a national level, and 502 centres of competence at a regional level. DM belongs to FILNEMUS, the neuromuscular RDs axis of the national plan.

The DM-scope concept was to create an integrative Information Technology (IT) platform providing tools to allow the collection of data during routine clinical management while promoting clinical research. A common core data set (CDS) appropriate for the DM population was created by collaboration between French and Canadian scientists. We also ensured that the CDS could enhance translational research in DM such as natural history studies, validation of outcomes measures and genotype-phenotype correlations. The DM-Scope system proposes specific tools to summarize clinical visit data and to help health-care practitioners in routine medical care.

This framework has been built to be shareable, interoperable and open to researchers and clinicians to favour a synergistic network in the DM community.

### Governance

DM-Scope was funded by *Association Française contre les Myopathies* (AFM)-Telethon patients association. Registry leadership consists of a co-ownership between AFM-Telethon and “*Assistance Publique-Hôpitaux de Paris*” (AP-HP), as well as an internal steering committee and an external scientific advisory committee. The internal steering committee includes principal and co-investigators, physicians and a DM representative from the patient group. The external scientific advisory committee consists of two expert scientists.

### Ethical and legal issues

DM-Scope registry has been approved by the French data protection authority (National Commission on Informatics and Liberty [CNIL]) (approval reference number: #1282122, date of approval: 2008). Patients are anonymously registered in a reversible way for practitioners only. The consent procedure was approved by the national ethic committee CCTIRS (Advisory Committee on Information Processing in Material Research in the Field of Health). The adult patients received an information letter and granted their verbal informed consent. The legal guardians and children received an information letter and legal guardians signed a written informed consent. The paediatric section was approved by Tours university hospital ethics committee (approval reference number: #2014–025, date of approval: 2014).

### Patient and data collection

DM-Scope registry collects relevant clinical and epidemiological data on a standardized form during routine medical evaluation performed in French neuromuscular reference centres. Only patients with confirmed genetic diagnosis were included and data was collected by health-care practitioners during their annual clinical visit. CDS is close to the common data element recommended by the EPIRARE project [11]. The DM-Scope form is shared with the Quebec registry to promote international research. The information is broadly divided into the following sections: demographic features (date of birth, gender, parental details, place of birth, place of residence, willingness to be contacted to participate in a future clinical study and date and reason of death), diagnosis, natural history (including description of the pregnancy, the newborn period, neurodevelopmental conditions for children), past medical history, education, social and professional impact of the disease, neuromuscular symptoms, orthopaedic deformations and facial dysmorphism for children, cardiac troubles, respiratory defects, digestive problems, endocrine dysfunction, current medications and interest for clinical research (current and past participation in clinical trials). Prioritisation of data collection is defined by a set of mandatory, prioritised and optional items.

**Fig. 1:**
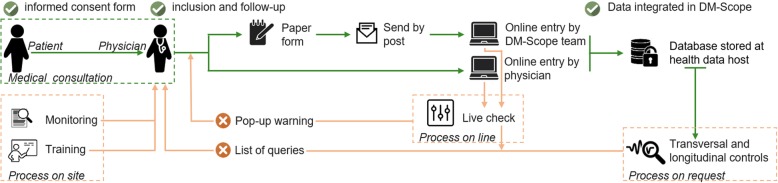
DM-Scope data processing and quality control

Health-care practitioners or clinical research assistants (CRA) input data either online into the DM-Scope system or onto a paper form received and entered by the curating centre (Fig. 1).

Patients can be informed about research studies, advances in knowledge of the disease and easily connected with expert neuromuscular centres. Information is available on the DM-Scope website (www.dmscope.fr). In a next step, patients should also request to be included in the registry using a self-recorded form to report data such as quality of life. This complementary enrolment allows the extension of the registration of DM individuals to patients who are not followed in RD reference centres.

### Security of data

Recorded data stored on secured file servers maintained by a clinical research unit at Montpellier hospital (France). This team is responsible for development, maintenance and the security of numerous registries and cohorts. The DM-Scope system consists of a robust security infrastructure to support authentication, confidentiality and data integrity. To access the system, every user is assigned a personal user name and password. The online input and access to the data are restricted to the practitioners or CRA who have a personal login. The access codes are generated by administrators, once the user has signed a written agreement. Health-care practitioners have an online and secure access to the data of the DM patients they follow. Patients are identified by the first three letters of their first and second name, gender and date of birth. However, only fully anonymous data are available to researchers and for analysis – all enrolled participants have a DM-Scope ID which is automatically generated.

In the near future, the registry plan to be connected with the national RD public health general registry (BAMARA) [45, 46].

### Data use and research applications

Investigators from expert centres are required to submit a research protocol to the internal steering committee. Data analysis and recruitment of patients starts once the project has been unanimously approved by the internal steering committee. All feasibility studies and identification of eligible patients for recruitment in clinical studies are performed by the coordinating centre staff. Publication policy and authorship composition are defined a priori. All-contributors are included in the authorship.

### Quality insurance procedures

Homogeneous data collection for new participants is ensured by an initial training program. CRA pays special attention to assess the quality of collected data and respect the standardized protocol. Each clinician is responsible for the content and quality of collected information.

Data input is controlled at three levels (Fig. 1). First, quality control occurs at the online input. Several data constraints have been integrated into the DM-Scope system which filter and generate automatic alerts when inconsistent data have been entered. For example, duplicate records are not allowed, consultation dates have to be superior to birth date, etc. In the case where data is entered by the coordinating centre staff, the automatically generated queries are sent to participating centres for resolution. Second quality control is performed on request, an R algorithm included in the DM-Scope platform allows a list of transversal and longitudinal inconsistencies and the generated queries sent to participant centres for resolution to be visualised. Last quality control is site monitoring. Regular visits to neuromuscular centres allow completion of undelivered data and clarify queries. Monitoring of collected data is facilitated by a specific tool created in the DM-Scope system. Furthermore, this platform provides a synopsis with complete and incomplete data per visit and per patient.

### Database architecture

DM-Scope registry is available on a secured website (www.dmscope.fr). Included tools are secure source applications with restricted data access to previously recorded professionals. Database is designed as web-server architecture and is accessible from anywhere at anytime. The statistical software R (version 3.5.0) is connected with the database.

The web-server was developed by 4D (version v17). 4D updates every 18 months. Daily backups are done by a program in the database settings. Each backup includes the structure and the data files for recovering the database automatically. The system is compliant with European Union’s General Data Protection Regulation (EU GDPR) and data are stored in an authorised system hosting personal health data.

### Functionalities and database interface

The interface layout has been designed to facilitate navigation and allows the use of various tools integrated into the system. Available functions vary according to the user type: general user, professional user and curators.

#### General user interface

The DM-Scope home page describes DM diseases, the DM-scope platform (including aims, guidelines, networks, research projects, underlying source documents, information links…), and news in the DM-field.

#### Professional interface

**Fig. 2:**
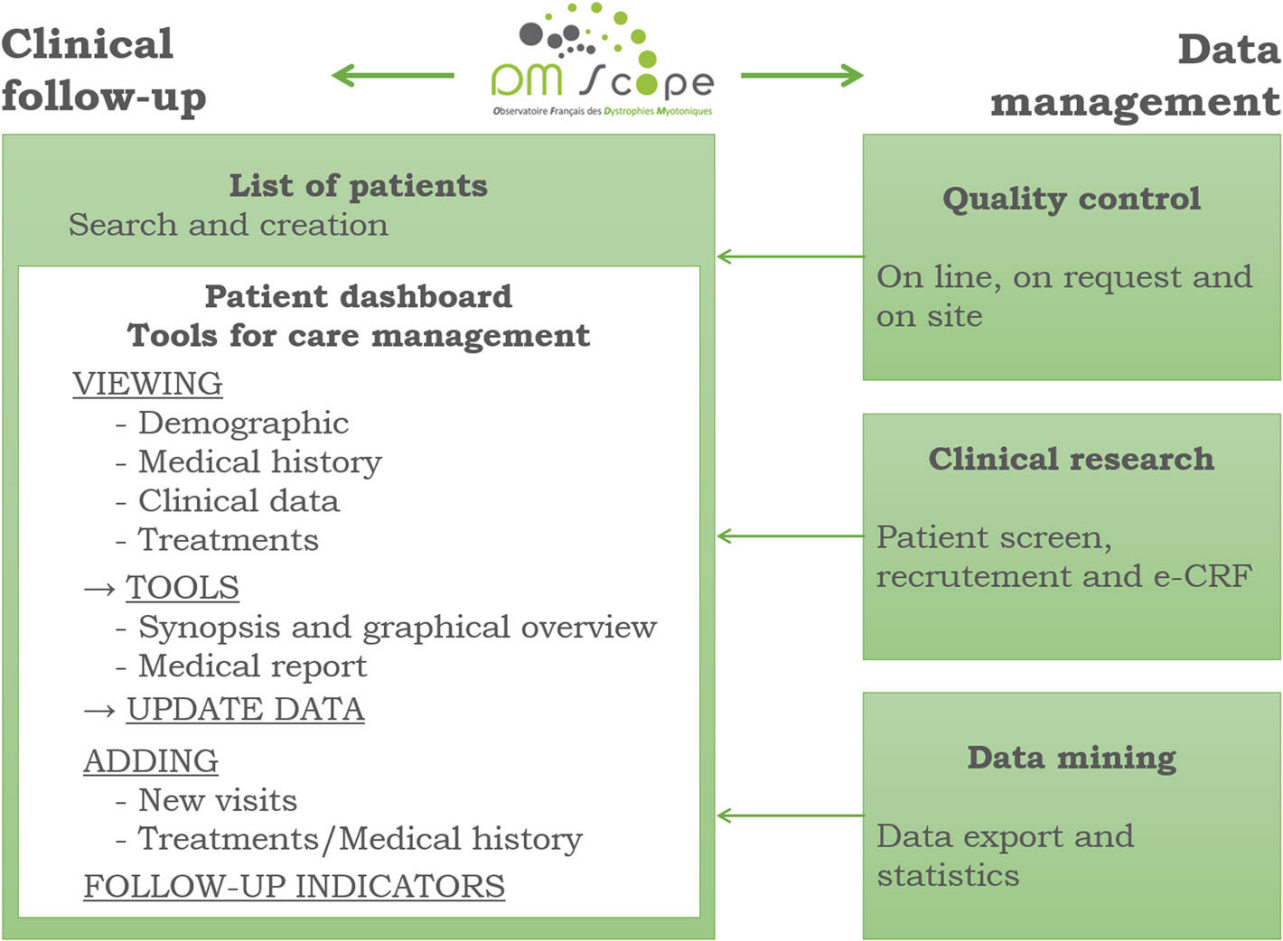
Functionalities and database interface

The DM-Scope system provides tools to optimize clinical evaluations (Fig. 2, left part). Home page for health-care practitioners includes a list of patients. Physicians or CRA can complete or create a new patient follow-up. Patient health is summarized in a dashboard that helps physicians to display a detailed overview of the collected longitudinal data, to edit synopsizes or medical reports, to visualize graphs following the severity of symptoms and refer to automatic index facilities. There is reciprocity between users and the coordinating centre to improve the data management system and enhance their intrinsic motivation.

#### Curator interface

In addition to displaying data in an organized manner, the curators have several tools to follow the network activity, to screen eligible patients for clinical studies and to identify patients with available biomaterials at AFM-Genethon Biobank. Supplementary functions allow summaries and graphic displays. Statistics and graphics are renewed at each DM-Scope update to report activity in each centre and to characterize the current French DM population.

### Statistical analyses

Cross-sectional analysis was performed using R 3.5.0 software (the R Foundation for Statistical Computing, Vienna, Austria). Descriptions are given in number and percentage N (%) for qualitative variables, in mean and standard deviation (SD) for quantitative variables or in median and interquartile range [Q1; Q3] in the case of a non-gaussian distribution. Missing data from subjects who had incomplete follow-up data were imputed using the Last-Observation-Carried-Forward method. Geographical distribution was presented using the cartography package (version 2.1.2) [47, 48]. Information on the French territorial departments and regions was acquired from the French National Geographic Institute (GEOFLA® 2.1). Sociodemographic data of the French population was based on French National Institute of Statistics and Economic Studies [49]. Survival analysis was performed using Kaplan-Meier curves. Date of inclusion in the study is defined as the date of the first symptom. The end date is the date of death or last follow-up. Cox proportional hazards models stratified by centres with a gamma frailty term is used to assess random effects across the contributing centres [50, 51].

## Results

### DM-scope registry activity

#### A nationwide coverage

**Fig. 3:**
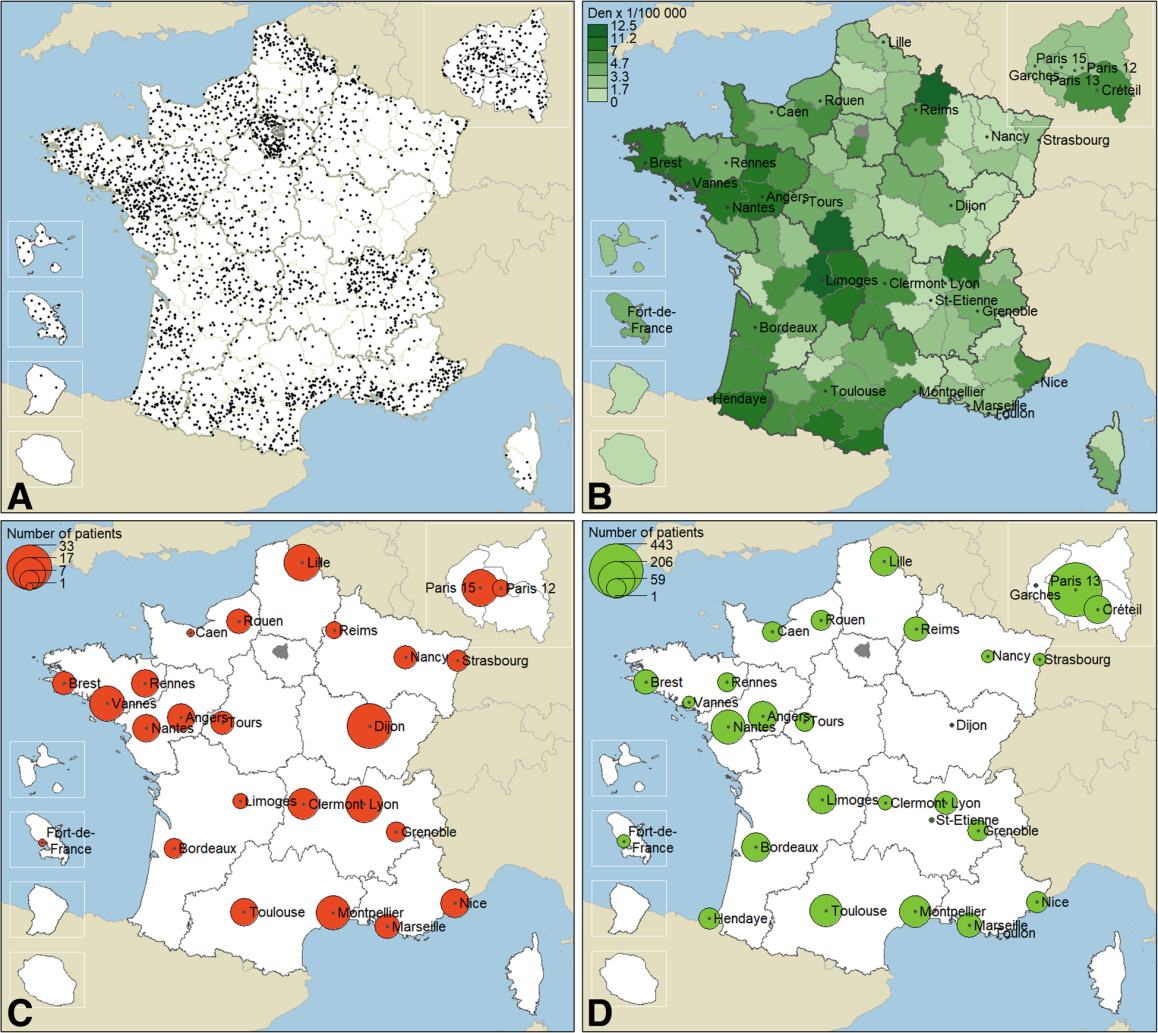
Cartography of place of residence of enrolled DM participants. **a** The individual representation (*N* = 2875). Each dot refers to one patient place of residence and dots position is allocated to a random position in the corresponding department (top left). **b** The regional distribution according to the density of population (N = 2875). Darker the green is, more the DM is prevalent in the department (top right). **c** Distribution of DM-Scope Registry enrolled patients among paediatric French neuromuscular expert centres (26 centres, *N* = 255). The number of enrolled patients is spot-size dependent (bottom left). **d** Distribution of DM-Scope Registry enrolled patients among adult French neuromuscular expert centres (29 centres, *N* = 2620). The number of enrolled patients is spot-size dependent (bottom right)

DM-Scope registry has a nationwide coverage (Fig. 3a). The distribution of DM patients is non-homogenous with a lower proportional representation of patients in the East of France since corresponding expert centres joined the registry more recently. The relative ratio of DM patients to the general population in each region of France was uneven. Five regions showed the highest density of DM patients (Fig. 3b): such as Limousin, Pays de la Loire, Champagne-Ardennes, Bretagne and Aquitaine.

Standardized data from DM enrolled patients were collected by 55 French RD expert centres (26 childhood and 29 adult centres). Fourteen of the 26 paediatric centres enrolled respectively more than 10 DM1 childhood patients (Fig. 3c). Half of the DM1 paediatric cohort was included by the 7 biggest centres: Dijon, Lille, Lyon (Bron), Paris (Necker), Vannes, Montpellier and Clermont-Ferrand. Thirteen of the 29 adult centres have more than 80 patients (Fig. 3d). Half of the adult cohort is managed by the 7 biggest adult centres: Paris (Pitié-Salpêtrière), Nantes, Toulouse, Montpellier, Angers, Bordeaux and Lille.

#### Regular enrolment of DM patients and annual data update

**Fig. 4:**
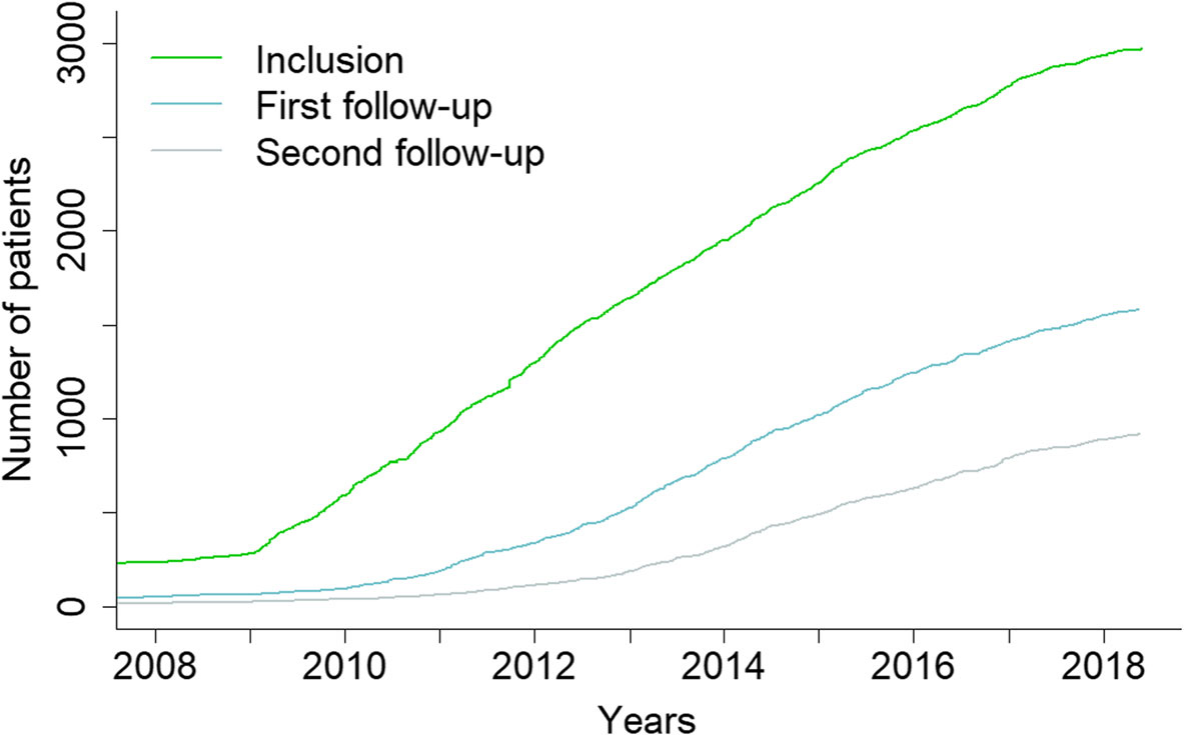
Cumulative number of participants in the DM-Scope Registry. The green line represents the number of included DM patients and the blue/grey line the number of first/second followed-up DM patients over time

The DM-scope registry has enrolled almost 3000 DM patients since 2008 (Fig. 4). Inclusion of the 2970 patients has been regular up to now (green line). The collected data have been annually updated since 2010. Between 2008 and 2018, more than half of the enrolled DM patients (53.3%) have been followed-up at least once, 30.9% at least twice, and 17.9% at least three times.

#### The DM-scope registry, a useful platform to promote DM research

DM-scope registry has facilitated the design, the recruitment of patients and the access to available biomaterials in various type of research studies (*n* = 10). Observational studies have led to refine the DM1 clinical classification [23], to identify gender as a modifying factor of the DM phenotype [52], altogether contributing to registry harmonization [53, 54] and to the definition of guidelines for medical care [55]. Other collaborative observational studies contributed to improve knowledge on myotonic stiffness in adults with DM1 [56], ophthalmologic defects [57], pyscho-cognitive aspects [58] and DM1 paediatric forms [59]. DM-scope registry also contributed to basic research by the identification of a unique interrupted genetic variant in two atypical DM1 pedigrees [60]. Finally, the DM-Scope registry was instrumental for the screening and recruitment of participants in interventional studies. One on-going study focuses on the impact of the early introduction of non-invasive ventilation [Clinical trial #NCT01225614]. A phase 2 pharmacological trial reported the benefit of metformin for locomotion [61]. In the European *Optimistic* clinical trial, the efficiency of the registry was evidenced by fast recruitment of 71 DM1 patients within a short timeframe (6 months) and a low screening failure rate [62, 63].

### DM-scope registry covers a well-characterized representative population

#### Demography (Table 1)

The DM-Scope registry includes 2828 DM1 patients (2506 adults and 322 children) and 142 DM2 adult patients. At the last visit, 62 DM1 children became adults and 95 patients died (86 DM1 adults, 5 DM1 children and 4 DM2 patients).

**Table 1:** Demographic characteristics, diagnosis and genetic of DM enrolled patients in DM-Scope registry

Variable	Level	DM1 (*N* = 2737)	DM2 (*N* = 138)	Total (*N* = 2875)
Demography				
Sex	Female	1453 (53.1%)	79 (57.2%)	1532 (53.3%)
Age at the last visit	mean (sd)	41.1 (16.0)	54.5 (14.2)	41.7 (16.1)
Adults	Age > 18 years	2482 (90.7%)	138 (100.0%)	2620 (91.1%)
Marital status	Single	1080 (47.3%)	34 (28.8%)	1114 (46.4%)
	*missing*	*200 (8.1%)*	*20 (14.5%)*	*220 (8.4%)*
Diagnosis and genetic				
Age of first symptoms	mean (sd)	23.5 (15.9)	38.1 (16.0)	24.2 (16.2)
	*missing*	*490 (17.9%)*	*24 (17.4%)*	*514 (17.9%)*
Age at clinical diagnosis, yrs	mean (sd)	32.5 (14.8)	48.3 (13.5)	33.4 (15.2)
	*missing*	*519 (19.0%)*	*9 (6.5%)*	*528 (18.4%)*
Age at molecular diagnosis, yrs	mean (sd)	33.4 (16.0)	50.6 (14.0)	34.3 (16.4)
	*missing*	*532 (19.4%)*	*13 (9.4%)*	*545 (19.0%)*
Delay between diagnosis, years	median [iqr]	8.6 [3.2, 17.2]	10.8 [4.4, 19.9]	8.9 [3.3, 17.4]
	*missing*	*1230 (44.9%)*	*40 (29%)*	*1270 (44.2%)*
Mutation size^a^	median [iqr]	550 [300, 900]	4000 [2750, 5000]	–
	*missing*	*584 (21.3%)*	*71 (56.5%)*	*–*
Transmission	Paternal	1100 (56.9%)	27 (39.7%)	1127 (56.4%)
	*missing*	*805 (29.4%)*	*70 (50.7%)*	*875 (30.4%)*

^a^CTG mutation for DM1 and CCTG mutation for DM2

Descriptions are given in number and percentage N (%) for qualitative variables; in mean and standard deviation (SD) or in median and interquartile range [Q1; Q3] for quantitative variables. Number of missing data is written in italic

In 2018, the registry counts 2876 living patients (2737 DM1 and 138 DM2). Demographic results revealed that women accounted for a slightly greater percentage of enrolled patients in both DM subtypes. The mean age of patients at the last visit is 41.1 yrs. (16.0) in DM1 and 54.5 yrs. (14.2) in DM2. 47.3% of DM1 patients compared to 28.8% of DM2 patients live alone.

#### Diagnosis and genetic characteristics

The clinical manifestations were the first causes of diagnosis in DM patients (47.5% for DM1 and 65.7% DM2). However, a substantial number of DM1 patients are diagnosed by familial genetic counselling (43.9% DM1 and 32.4% DM2). Only few patients, exclusively DM1, were identified after the occurrence of a child with a congenital form (7.2% DM1). Diagnoses were made on average at 32.5 yrs. (14.8) in DM1 and 48.3 yrs. (13.5) in DM2 for clinical diagnosis and on average at 33.4 yrs. (16.0) in DM1 and 50.6 yrs. (14.0) in DM2 for molecular diagnosis *(*Table 1*)*. The delay between the first symptom and the molecular diagnosis is on average at 8.5 yrs. [3.0, 17.0] in DM1 and 10.8 yrs. [4.4, 19.9] in DM2.

Genetic tests were available in 77.4% of diagnosed patients. The median mutation size was 550 [300, 900] (min-max: 41–5000) CTG repeats in DM1 and 4000 [2750, 5000] (min-max: 185–23,100) CCTG repeats in DM2. In contrast with DM2, where the transmission is mainly maternal (60.3% of transmissions), DM1 is more often transmitted by the father (56.9% of transmissions).

#### Clinical spectrum

The DM-Scope registry covers a large clinical spectrum as previously described [13]. On average, the first symptom generally appeared at the age of 23.5 (15.9) in DM1 and at the end of the third decade in DM2 (38.1 yrs. (16.0)). Disease onset occurred over a very large age range (min-max: 0-73 yrs. in both types). The French DM1 population included the five clinical forms classified on the basis of age at onset: congenital (onset < 1 month; 230 (9.0%)), infantile (onset between 1 month and 10 yrs.; (424 (16.5%)), juvenile (onset between 11 yrs. and 20 yrs.; (724 (28.2%)), adult (onset between 21 yrs. and 40 yrs.; 810 (31.6%)) and late form (onset after 40 yrs.; 376 (14.7%)).

#### Education and employment (Table 2)

Only DM1 adult patients were still students (4.5% adult DM1, 0% adult DM2). The mean age at the end of education was similar in the two DM subtypes (18.8 yrs. (3.9) in DM1 and 18.9 yrs. (3.9) in DM2). DM1 patients were more frequently schooled in specialized educational conditions (14.6% adult DM1, 2.3% adult DM2). The proportion of DM2 patients having an educational level ISCED> 3 was higher than in DM1 (51.8% in DM2, 39.2% in adult DM1).

**Table 2:** Educational and employment of DM enrolled patients in the DM-Scope registry

	Level	DM1		DM2	All
		Children	Adults		
		(*N* = 255)	(*N* = 2482)	(*N* = 138)	(*N* = 2875)
Education					
In education at the last visit	Yes	24 (96.0%)	97 (4.5%)	0 (0.0%)	121 (5.3%)
	*missing*	*230 (90.2%)*	*336 (13.5%)*	*42 (30.4%)*	*608 (21.1%)*
Age at education end, years	mean (sd)	–	18.8 (3.9)	18.9 (3.9)	18.8 (3.9)
	*missing*	*255 (100%)*	*1819 (73.3%)*	*99 (71.7%)*	*2173 (75.6%)*
Educational environment	Specialized	80 (40.4%)	284 (14.6%)	2 (2.3%)	366 (16.5%)
	*missing*	*57 (22.4%)*	*543 (21.9%)*	*52 (37.7%)*	*652 (22.7%)*
Final education on ISCED scale	level > 3	5 (3.4%)	729 (39.2%)	43 (51.8%)	777 (37.2%)
	*missing*	*107 (42%)*	*622 (25.1%)*	*55 (39.9%)*	*784 (27.3%)*
Employment					
In activity at the last visit	Yes	–	654 (27.6%)	27 (25.0%)	681 (27.5%)
	*missing*	–	*111 (4.5%)*	*30 (21.7%)*	*141 (4.9%)*
Time of activity	Part-time	–	201 (34.2%)	7 (28.0%)	208 (33.9%)
	*missing*	–	*66 (2.7%)*	*2 (1.4%)*	*68 (2.4%)*
Professional environment	Specialized	–	137 (27.6%)	1 (4.5%)	138 (26.6%)
	*missing*	–	*158 (6.4%)*	*5 (3.6%)*	*163 (5.7%)*
Reason for unemployment	Due to the disease	–	933 (70.8%)	24 (35.8%)	957 (69.1%)
	*missing*	–	*399 (16.1%)*	*14 (10.1%)*	*413 (14.4%)*

Descriptions are given in number and percentage N (%) for qualitative variables; in mean and standard deviation (SD) for quantitative variables. Number of missing data is written in italic

Only one third of DM adults were employed at time of last visit (27.6% in adult DM1, 25.0% in DM2). DM1 individuals had a part-time employment more frequently than those with DM2 (34.2% of DM1 adults, 28.0% of patients in DM2). Among DM1 active adults, 27.6% worked in a specialized professional environment, and only one among DM2 patient. Unemployment is mainly due to the disease (70.8% in DM1; 35.8% of DM2). All social-professional categories were represented in the two DM forms. More than one half of the DM active adults were employees or workmen (68.9% in DM1, 57.6% in DM2), 16.7% of DM patients worked in intermediate professions (16.7% in DM1, 18.2% in DM2) and few DM patients had positions with responsibilities, such as shopkeepers or company heads (4.4% in DM1, 6.1% in DM2). DM2 patients were more represented in executive and intellectual professions (9.9% in DM1, 18.2% in DM2).

#### Survival analysis with heterogeneity between centres

**Fig. 5:**
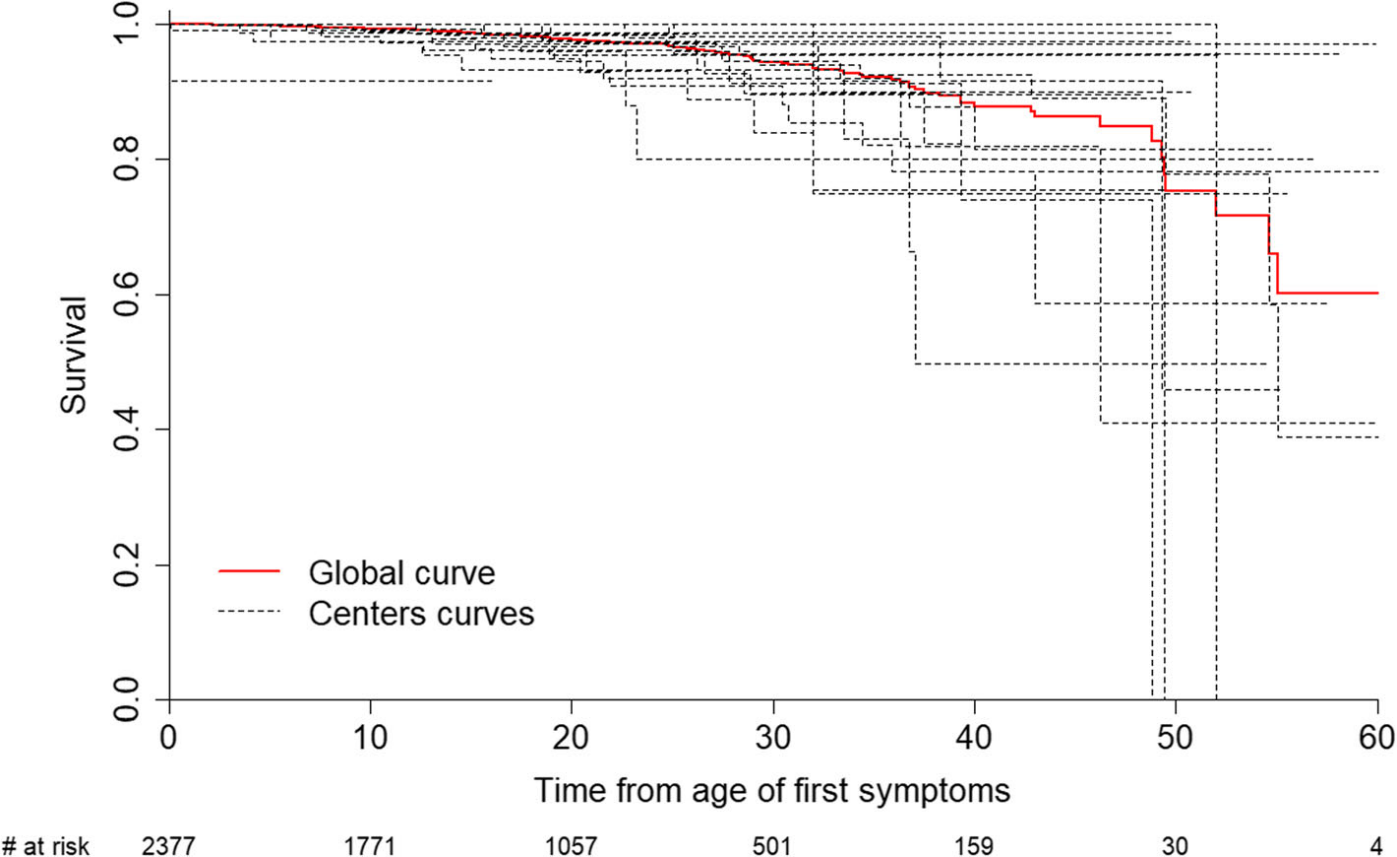
Kaplan-Meier curves for all-cause mortality. The red line indicates the survival of the overall registry DM population; the dark dotted lines represent survival of subgroups in the 33 individual neuromuscular centres (only centres including more than 10 patients are selected)

Survival analyses were performed on 1476 patients and 92 events. The Kaplan-Meier survival curve for the total cohort is illustrated in Fig. 5. Median follow-up is 17.4 [9.6; 28.0] years, the probability of survival at 30 years is 0.94.

Among the 55 centres, 33 centres did not report any death cases and 7 centres recorded more than 5 dead patients. Survival analysis is performed on 33 centres (≥10 patients). Figure 5 shows the heterogeneity between the 20 centres which recorded death status and the variance of random effects is 0.22.

## Discussion

This report describes the DM-Scope registry, an innovative concept that overcomes most of RD registries limitations. Indeed, health authorities pointed to main limitations of RD registries including underreporting of outcomes, missing data, and/or inadequate follow-up. Robustness of gathered data depends on the quality of data entry, the number of enrolled patients, the diversity of their demographic and disease characteristics, including age-annotated manifestations, and the retention of recruited patients [1–4].

DM-Scope registry overall model is based on (1) an innovative IT platform that provides tools for clinicians to facilitate the management of DM patients, and on (2) the network of neuromuscular expert centres established by the national RDs plan in France. The registry federates RD expert physicians, from 55 French neuromuscular expert centres, and promotes a longitudinal standardised data collection. To our knowledge, such platform is a unique example that helps to optimize medical care as well as facilitate research in RD. By enabling the input of multidisciplinary expert physicians and limiting the contribution of cognitively impaired DM patients, this registry ensures highest quality of data. While other DM registries have been established [64] the DM-Scope registry is the largest one with almost 3000 DM enrolled patients accounting for more than 20% of overall registered DM patients internationally [53]. Furthermore, the registry collects the whole range of demographic and phenotypic characteristics of this RD condition. Indeed, standardized data span from congenital patients at birth to late onset adult patients. In addition, the platform includes three levels-quality insurance procedure.

The registry coverage is nationwide though some regions are under-represented. This is likely related to the activity of the neuromuscular expert centres and more recent partnered centres should homogenise the national distribution in the future. Studies to assess the DM prevalence are limited [13] and the exact prevalence in France is unknown. In addition, the DM disease is not listed for genetic screening in most countries. Therefore national coverage of our registry contributes to estimate the distribution of DM individuals and regional differences. Some differences were observed in the relative distribution of DM patients according to the general population density in some geographic areas suggesting that the prevalence of DM is uneven across France. For example, in the Basque region a high DM1 frequency was observed which is consistent with the report by López de Munain et al. [45]. To confirm such regional disparities in France, we plan to analyse complementary data from the national BAMARA registry [46]. It should be noted that DM-Scope and BAMARA registries are not designed for prevalence studies since they are not intended to collect the complete disease population.

Recently, we decided to record death status which allows (1) to estimate the severity of the disease; (2) to minimise bias in cross-sectional studies due to loss of follow-up due to death; (3) for patient screening and enrolment in clinical trials; (4) to assess various prognostic factors of death. Survival analyses in DM are scarce with no recent population cohort data existing. Our results showed an annual frequency of death consistent with previous reports [65]. In our case, the number of deceased patients is likely under-estimated since the vital status recording was more recently introduced leading to no record of death being reported by many centres. An accurate identification of death is limited since it is not part of annual clinical follow-up management. We expect to further improve the registry by identifying patients not seen (> 3 years) by clinician determination of the patient status: lost to follow-up vs death. In addition, complementary analyses from administrative national databases [66] will significantly improve survival estimations.

As part of the national RD plan, the DM-Scope registry will allow longitudinal comparison of medical practice between RD expert centres with the purpose of promoting a harmonization of DM medical care nationally as well as contributing to healthcare guidelines for DM.

The DM-Scope Registry covers the large clinical and genetic spectrum of DM patients [14, 16] with the representation of all social and professional conditions. The registry provides opportunities to characterize large DM cohorts of adults or children, to clarify genotype-phenotype correlations, to study the social and professional consequences of DM as well as to compare the DM1 and DM2 genetic entities. While encompassing all disease organ and system involvement, the registry currently lacks items describing the cognitive impairment. Over the past few years, international workshops [67, 68] have focused on how to assess central nervous system involvement. Some time-consuming neuropsychological tests are currently discussed and require validation for future integration into registry dataset. Missing data are mainly related to optional items and seem randomly distributed.

The DM-Scope registry has drawbacks including (1) the lack of items related to the cognitive impairment, (2) the underreporting of deceased cases, and (3) missing data.

Our platform has already proven to be a key instrument for promoting clinical studies and generating data for medical care guidance in DM1. In fact, the registry substantially facilitated DM translational research by (1) refining the DM1 clinical classification; (2) accessing to available biomaterials for molecular basic research studies; (3) the design and the recruitment of patients in both observational and interventional studies; and (4) producing evidence-based material for care guidelines in adult and childhood DM populations. Future longitudinal analyses from the DM-Scope registry will be conducted to refine the clinical characteristics of the DM population.

The transferable strengths of the registry rely in the fact that it is a shareable and interoperable framework which promotes multicentre high quality data collection in a large population. In this way, the DM-Scope registry has recently evolved into an international consortium (named iDM-Scope) to harmonise the French and Quebec cohorts. Such data standardization allows the comparison of DM characteristics in two different populations. Data harmonization helps to improve translational research including natural history studies, biomarker identification and outcome measures and facilitates the recruitment of patients in upcoming transnational multicentre trials. It represents a first step to contribute to cross-border healthcare in DM. In addition, DM-scope concept can serve as a model to other RDs.

## Conclusion

The DM-scope registry overcomes some of the main challenges of RD registries. By facilitating the contribution of clinicians and creating a standardized data collection, our system provides robust data nationwide. The link between clinical features, genotype, available biomaterial and trial datasets, creates this platform a powerful device for the harmonization of international DM network activities and for the design of multicentre studies. The DM-Scope registry has been proven effective for various translational research studies and also in clinical trials. Finally, the DM-scope concept can serve as a generalizable model to other countries and to other rare diseases.

## Abbreviations

AFM: “Association Française contre les myopathies”
AP-HP: “Assistance Publique des Hôpitaux de Paris”
BAMARA: National Rare disease Public Health general Registry
CCTG: Cytosine, Cytosine, Thymine, Guanosine
CCTIRS: “Comité consultatif sur le traitement de l’information en matière de recherche”
CDS: Core Data Set
CNIL: *“Commission nationale de l’informatique et des libertés”*
CRA: ClinicalResearch Assistant
CTG: Cytosine, Thymine, Guanosine
DM: Myotonic Dystrophy
DM1: Myotonic Dystrophy type I
DM2: Myotonic Dystrophy type II
EPIRARE: European Plateform for Rare Disease Registries Project
EU GDPR: European Union’s General Data Protection Regulation
ISCED: International Standard Classification of Education
IT: Information Technology
RD: Rare diseases
RDR: Rare disease Registries
SD: Standard deviation
